# Low Interleukin - 8 Level Predicts the Occurrence of the Postpericardiotomy Syndrome

**DOI:** 10.1371/journal.pone.0108822

**Published:** 2014-10-21

**Authors:** Maria Jaworska-Wilczyńska, Adriana Magalska, Katarzyna Piwocka, Piotr Szymański, Mariusz Kuśmierczyk, Maria Wąsik, Tomasz Hryniewiecki

**Affiliations:** 1 Department of Valvular Heart Disease, Institute of Cardiology, Warsaw, Poland; 2 Department of Cardiac Surgery and Transplantology, Institute of Cardiology, Warsaw, Poland; 3 Nencki Institute of Experimental Biology Polish Academy of Sciences, Warsaw, Poland; 4 Department of Laboratory Diagnostics and Clinical Immunology of Developmental Age, Medical University of Warsaw, Warsaw, Poland; University of Bologna, Italy

## Abstract

**Aims:**

The objective of this study was to investigate inflammatory markers of the postpericardiotomy syndrome (PPS) and to determine individuals prone to develop the PPS.

**Methods and Results:**

The study included 75 patients with a stable coronary disease that had underwent coronary artery bypass surgery. Serum samples were collected prior to the surgery and on the 5th day after the operation, to measure the concentration of IL-8, IL-6, IL-1β, IL-10, TNF, IL-12p70. All included patients were screened for the PPS before discharge from the hospital and 6 months after the surgery. The 49 patients developed the PPS (65.4%), among them 42 (56%) patients had pleural effusion, and 23 (31%) had pericardial effusion. The cytokine analysis has shown an inverse correlation between IL-8 concentration before the surgery, and the occurrence of the PPS (p = 0.026). There were also positive correlations between the magnitude of increase of IL-8 and IL-1β concentrations on the 5th day after the surgery and the occurrence of the PPS (p = 0.006 and p = 0.049 respectively). Multivariate analysis revealed IL-8 concentration before surgery as an independent risk factor of the PPS development (HR = 0.976; 95%CI: 0.956–0.996, p = 0.02). Cut-off point was established to assess the predictive value of IL-8 concentration (21.1 pg/ml). The test parameters were: sensitivity: 62.5%, specificity: 75%, positive predictive value: 83% and negative predictive value: 50%. Clinical evaluation showed the relationship between the hemoglobin concentration before the surgery and the PPS occurrence (p = 0.01).

**Conclusion:**

The IL-8 and IL-1β may participate in the postpericardiotomy syndrome pathogenesis, and the IL-8 concentration measurement may select patients with the risk of the PPS development.

## Introduction

The postpericardiotomy syndrome (PPS) is an inflammation of the pericardium or pleura following a variety of pericardial injuries [1]. It was described to occur after cardiac surgery, acute myocardial infarction, percutaneous interventions, such as radiofrequency catheter ablation, coronary artery percutaneous interventions, implantation of cardiac pacemakers, or after thoracic surgery or trauma [2]–[8].

The PPS develops within weeks or months after pericardial injury [9]. The diagnosis of the PPS is based on clinical symptoms. There is a lack of diagnostic tests to expect the PPS. The clinical features of the PPS include low-degree fever, leukocytosis and elevated erythrocyte sedimentation rate, and increased C-reactive protein levels. The major symptoms are retrosternal or left precordial chest pain, non-productive cough and dyspnea. In examination, a pericardial or a pleuritic friction rub may be present [10].

The PPS is a common complication after surgery procedures, estimated to affect 10–40% of cases [9], [11]. The diagnosis of the PPS leads to prolonged hospitalization, possibility of relapses, and/or to severe complication such cardiac tamponade, constrictive pericarditis or coronary artery bypass graft occlusion [1], [9], [11].

The pathogenesis of the PPS is poorly understood. It is believed that injury of mesothelial pericardial cells and blood presence in the pericardial space trigger inflammation, with pericardial and pleural effusion, and systemic inflammatory response [1], [12]. Patients suffering from the PPS produce greater amount of anti-heart antibodies [13]. There is suggestion that autoimmune processes underlie the PPS development [14], [15].

The objective of this study was to investigate early inflammatory markers (cytokines) described previously to participate in inflammation after cardiac surgery [16]–[19], both pro-inflammatory: IL-8, IL-6, TNF, IL-1β, IL-12p70 and anti-inflammatory: IL-10. Furthermore we determined individuals prone to develop the PPS, and established whether the surgery method with or without extracorporeal circulation (ECC+ or ECC−) is related to the PPS development.

## Materials and Methods

The study was approved by Ethics Committee of Institute of Cardiology, permit number 2.51/11/10. The patients gave their informed written consent to participate in this study. Seventy five patients, 59 males and 16 females with stable coronary artery disease, undergoing coronary artery bypass grafting (CABG), were included into the study. The study selected consecutive patients.

The mean age was 65.31 years. The pre-operative data of the patients are shown in table 1. Diabetes and hypercholesterolemia were defined as newly found elevated glucose or cholesterol level, respectively, or currently treated disease. Chronic renal disease was defined as GFR<60 ml/min/1.73 m^2^. Tobacco use history was defined as current smoking, or smoking during the last year.

**Table 1:** Baseline characteristic of the studied population.

Variable	Number of patients (%)	Mean (SD)	Median
Age (years)		65.31 (9.26)	65
Female gender	16 (21)		
Male gender	59 (79)		
BMI (kg/m^2^)		27.51 (3.59)	26.8
Hb before CABG (g/dl)		13.58 (1.64)	13.9
Hb after CABG (g/dl)		11.11 (1.26)	10.8
Surgery duration (h)		3.79 (1.27)	3.5
Numbers of by-passes		2.32 (0.88)	2
MI history	33 (44)		
Chronic Renal Disease	20 (27)		
Diabetes Mellitus	23 (31)		
Hypercholesterolemia	44 (59)		
Tabacco use	14 (19)		
Hypertension	58 (77)		
HF (EF<60%)	33 (44)		

BMI-Body Mass Index, Hb-Hemoglobin, CABG-Coronary Artery Bypass Graft, MI-myocardial infarction, HF-Heart Failure, EF-Ejection Fraction, SD- Standard Deviation.

For the analysis we excluded subjects with acute coronary syndrome, surgery other than CABG, cancer, known chronic inflammatory diseases, pericardial or pleural effusion before the surgery, severe renal or liver insufficiency, and with local or systemic infection.

The postpericardiotomy syndrome was diagnosed according to published Colchicine for the Prevention of the Post-Pericardiotomy Syndrome trial (COPPS) criteria [20], before the discharge from the hospital, and/or by phone call at six month after the surgery. The diagnosis of the PPS was based on the presence of two or more findings: fever without evidence of infection, pleuritic chest pain, friction rub, evidence of pleural or pericardial effusion.

The day before surgery, and on the 5th postoperative day, 3 ml of venous blood sample was obtained. Immediately, the blood sample was placed into the tube without an anticoagulant. After blood clotting, the serum was separated by centrifugation (30000 g for 10 min), transferred into clean tubes, and then stored at –20°C for cytokine analysis. The BD Cytometric Bead Array Human Inflammatory Cytokines Kit (Becton-Dickinson) was used to quantitatively determine the pro-inflammatory cytokines IL-8, IL-1β, IL-6, TNF-α, IL-12p70, and anti-inflammatory IL-10, concentrations in the samples of serum or plasma. The preparation of the beads, standards, specific antibodies, reagents and serum samples, as well as protocols for flow cytometer setup, data acquisition and analysis, were performed according to Becton-Dickinson original instruction that was attached to the kit. Briefly, six bead populations with distinct fluorescence intensity have been coated with PE-conjugated antibodies (FL2) specific for the above mentioned cytokines. Fifty µl of mixed capture beads coated by specific antibodies were transferred to each assay tube and mixed together with 50 µl of recombinant standards, or patients' serum samples diluted according to the manufacturer's recommendations. Then the tubes were incubated for 1.5 h at room temperature, protected from light. After incubation, the samples were washed with 1 ml wash buffer, and centrifuged for 5 min at 200 g. The PE Detection Reagent (50 µl/test) was added to the 100 µl residual volume that had been leaved in each tube. After 1.5 h incubation with protection from light, the samples were washed once again with 1 ml of wash buffer, centrifuged (5 min, 200 g), mixed with 300 µl of wash buffer, and subjected to the cytometric analysis using cytometer Cytomix FC500 or Epics XL (Beckman-Coulter Co, San Diego, CA, USA), or FACSCalibur (Becton Dickinson, Franklin Lakes, NJ, USA). The cytometers possessed the argon laser (488 nm) and hel-neone (633 nm) or diode (635 nm) lasers.

Continuous variables were reported as mean or median. Discrete variables were given as frequency or percent. Normal distribution of the data was determined by Kolmogorov–Smirnov test. Clinical data and cytokines were analyzed using Students *t*-test for results with Gaussian distribution, non-parametric: sign test, Wilcoxon paired test, Mann-Whitney U test for two groups of non-dependent results, nonparametric sign test for two groups of dependent results, and Chi-square test (χ^2^) with Yates correction for small number of cases. *P* value<0.05 was considered as statistically significant. Linear regression analysis was performed to investigate the multivariable-adjusted association of PPS with clinical features or cytokines. Cut-off point at ROC (Receiver Operating Characteristic) was established to assess predictive value of IL-8 concentration. The test parameters sensitivity, specificity, positive and negative predictive value were performed.

All calculations were performed using SAS software version 9.2.

## Results

The PPS was diagnosed in 49 patients (65.4%), among them 42 (56%) patients had pleural effusion, and 23 (31%) had pericardial effusion. The diagnostic criteria for the PPS were shown in table 2.

**Table 2:** Diagnostic criteria for the PPS.

Event	Number of patients	% (of all patients)
Fever beyond first post-operative week	12	(24%)
Pericardial effusion	23	(31%)
Pleural effusion	42	(56%)
Friction rib	5	(7%)
Pleuritic chest pain	32	(42%)

There was no significant correlation between the occurrence of the PPS and age, sex, BMI, the surgery duration, the number of coronary artery bypasses, postoperative hemoglobin levels. There was also no link between the PPS and the transfusion of red blood cells in the postoperative period, hypertension, hyperlipidemia, heart failure with reduced ejection fraction, myocardial infarction in the past, diabetes mellitus, chronic kidney disease, and smoking (*Table 3*). Statistical analysis performed using Chi-square test (χ^2^) showed no increased risk for PPS and the type of surgery with or without extracorporeal circulation (ECC+ or ECC−) (p = 0.23).

**Table 3:** Comparison of clinical events between patients with and without PPS.

Clinical Feature	PPS (n = 49)	No PPS (n = 26)	P Value
Age [years] mean (SD)	66.54 (8.14)	64.75 (9.81)	0.16
Age [years] median	66	62.5	
Gender			
Female [number (%)]	12 (75%)	4 (25%)	0.16
Male [number (%)]	37 (63%)	22 (37%)	
BMI [kg/m^2^]			
mean (SD)	27.79 (3.53)	27 (3.65)	0.39
median	26.97	26.46	
Surgery duration [h]			
mean (SD)	3.97 (1.28)	3.48 (1.2)	0.12
median	3.5	3.5	
Number of by-passes			
mean (SD)	2.35 (0.85)	2.27 (0.94)	0.72
median	2	2	
Hb before surgery [g/dl]			
mean (SD)	13.23 (1.7)	14.25 (1.27)	0.01
median	13.7	14	
Hb after surgery [g/dl]			
mean (SD)	11.15 (1.36)	11.02 (1.03)	0.78
median	11	11	
Type of surgery			
ECC+ [number (%)]	14 (28.6)	11 (42.3)	0.23
ECC− [number (%)]	35 (71.4)	15 (57.7)	
Hypertension [number (%)]	38 (77.6)	20 (76.9)	0.21
Diabetes mellitus [number (%)]	16 (32.7)	10 (38.5)	0.18
Hypercholesterolemia [number (%)]	27 (55.1)	17 (65.4)	0,12
Heart failure EF<60% [number (%)]	19 (38,8)	14 (53,8)	0,1
Chronic Renal Disease [number (%)]	15 (30,6)	6 (23)	0,17
MI history [number (%)]	20 (40,8)	13 (50)	0,16
Tobacco use [number (%)]	8 (16)	6 (23)	0,12
Red Blood Cells Transfusion [number (%)]	30 (61)	13 (50)	0,33

BMI-Body Mass Index, Hb-Hemoglobin, ECC- extracorporeal circulation, CABG-Coronary Artery Bypass Graft, MI-myocardial infarction, HF-Heart Failure, EF-Ejection Fraction, SD- Standard Deviation, PPS-patients with the postpericardiotomy syndrome, No PPS- patients without the postpericardiotomy syndrome.

However, the lower preoperative hemoglobin level was a risk factor for the PPS development (*Table 3*).

A model of multivariate logistic regression analysis with stepwise variable selection option (“stepwise”) to determine independent predictors of the PPS occurrence identified the hemoglobin concentration before surgery (hazard ratio 0.643, 95% confidence interval 0.247 to 1.673, p = 0.01).

Analysis of cytokine concentration showed an inverse relationship between IL-8 concentration before the operation and the incidence of PPS (p = 0.026). Analysis of other cytokines showed only a tendency to the higher frequency of PPS in patients with the lower concentrations: IL-1β (p = 0.056), IL-10 (p = 0.07) and TNF (p = 0.077) before the surgery (*Table 4*).

**Table 4:** Analysis of cytokine concentration.

Cytokine	Time of blood collection	No PPS Median [pg/ml] (Q_1_–Q_3_)	PPS Median [pg/ml] (Q_1_–Q_3_)	P value
IL-8	Before surgery	32.27 (17.62–57.45)	17.2 (10.77–34.8)	0.026
	After surgery	59 (46.54–75.23)	45.99 (33.4–90.3)	0.63
IL-1β	Before surgery	15.3 (0–19.3)	0 (0–15.9)	0.056
	After surgery	7.66 (0–19.3)	2.2 (0–21.15)	0.8
IL-6	Before surgery	12.93 (6.45–26.2)	8.86 (3.88–18.89)	0.24
	After surgery	38.4 (31.85–50.5)	41.06 (30.92–79.1)	0.42
IL-10	Before surgery	17.92 (1.51–28.4)	1.61 (1.05–19.6)	0.07
	After surgery	9.87 (1.63–32.75)	3.6 (1.51–31)	0.59
TNF	Before surgery	16.57 (0–28.8)	0 (0–23.5)	0.077
	After surgery	14.46 (0–37.68)	1.43 (0–33.05)	0.67
IL-12p70	Before surgery	17.36 (0–32.6)	0 (0–25.2)	0.12
	After surgery	13.19 (0–34.03)	1.89 (0–30.7)	0.55

PPS-patients with the postpericardiotomy syndrome, No PPS- patients without the postpericardiotomy syndrome.

In order to determine the independent prognostic factors for the occurrence of the PPS, multivariate logistic regression model with stepwise variable selection option (“stepwise”) was performed. Among the variables examined: IL-8 before the operation, difference in the concentrations of IL-8 before and after surgery (ΔIL-8), IL-6, difference in concentrations IL-6 before and after surgery (ΔIL-6), IL-10, difference in concentration of IL-10 before and after surgery (ΔIL-10), TNF before the CABG, and the difference in concentrations TNF before and after surgery (ΔTNF), IL-1β before the operation, and the difference in concentration of IL-1β before and after surgery (ΔIL-1β), only IL-8 concentration before the surgery was identified as a risk factor of PPS (hazard ratio 0.976, 95% confidence interval 0.956 to 0.996, p = 0.02).

**Figure 1:**
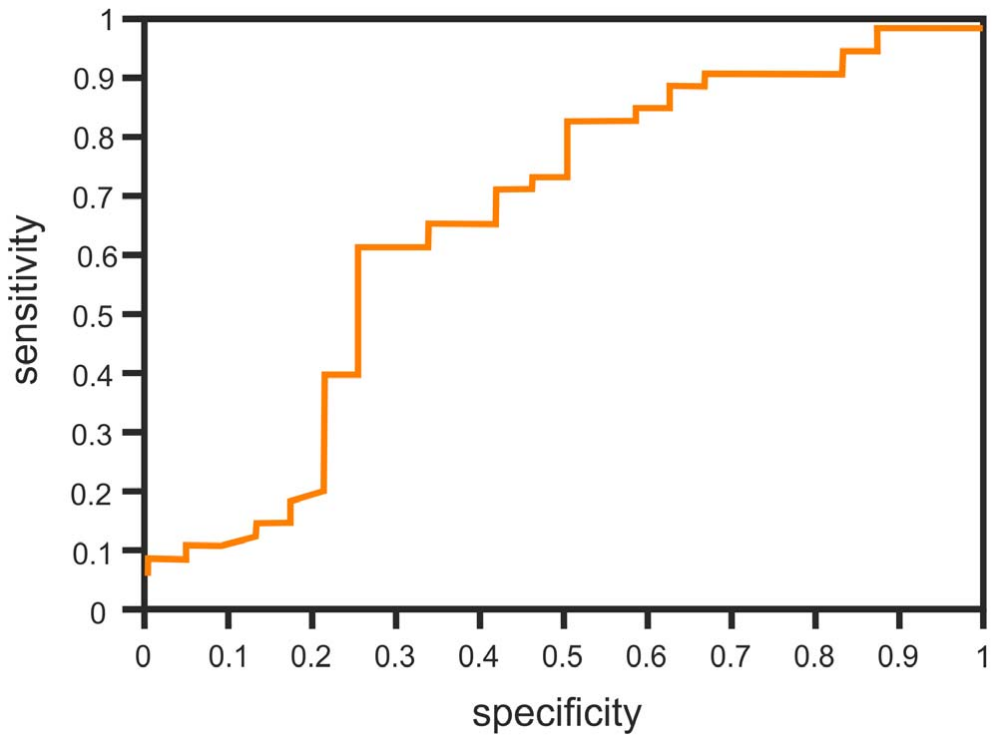
The ROC of IL-8 concetration before the surgery.

The cut-off point with maximum sensitivity and specificity of the test variable was estimated. The cut-off point for IL-8 levels before surgery was 21.1 pg/ml. When the concentration of IL-8 before surgery dropped by 1 pg/ml, a chance of PPS increased by 2.3% (hazard ratio 0.977, 95% confidence interval 0.957 to 0.997, p = 0.02) (*Figure 1*).

The analysis of the changes in cytokine concentrations collected 5 days after and prior to the surgery in individual patients without the PPS, by sign test, showed a statistically significant change in concentrations of IL-8 and IL-6 (p = 0.04 and p = 0.0005 respectively). In the group of patients diagnosed with the PPS in the follow up, all tested cytokines concentrations were statistically significant (*Table 5*). However, comparison of changes in cytokine levels in patients who developed the PPS to the patients without the PPS, by Wilcoxon test, revealed a significant difference for the concentration of IL-8 (ΔIL-8, p = 0.006) and IL-1β (ΔIL-1β, p = 0.049) as shown in table 5.

**Table 5:** The analysis of changes in cytokines concentration after and before surgery.

Changes in the cytokines concentration	PPS (n = 49)		No PPS (n = 26)		P value*
	Median (Q1–Q3)	P value	Median (Q1–Q3)	P value	
IL-8	33.3 (17.3–50.6)	<0.0001	13.5 (8.43–23.8)	0.04	0.006
IL-1β	1.82 (0–6.4)	0.001	0 ([−3.3]–4.9)	0.79	0.049
IL-6	27.56 (16.7–60.1)	<0.0001	21.93 (13.9–40.3)	0.0005	0.22
IL-10	1.63 (0–9.9)	<0.0001	1.18 ([−0.7]−3)	0.16	0.21
TNF	0.3 (0–9.16)	<0.0001	0 (0–4.75)	0.33	0.3
IL-12p70	0 (0–6.66)	<0.0001	0 (0–0.9)	0.65	0.27

PPS-patients with the postpericardiotomy syndrome, No PPS- patients without the postpericardiotomy syndrome, P value-changes of cytokines after and before surgery tested by sign test, P value*- the differences of cytokine changes in patents with and without PPS tested by Wilcoxon test, n- numbers of patients.

**Figure 2:**
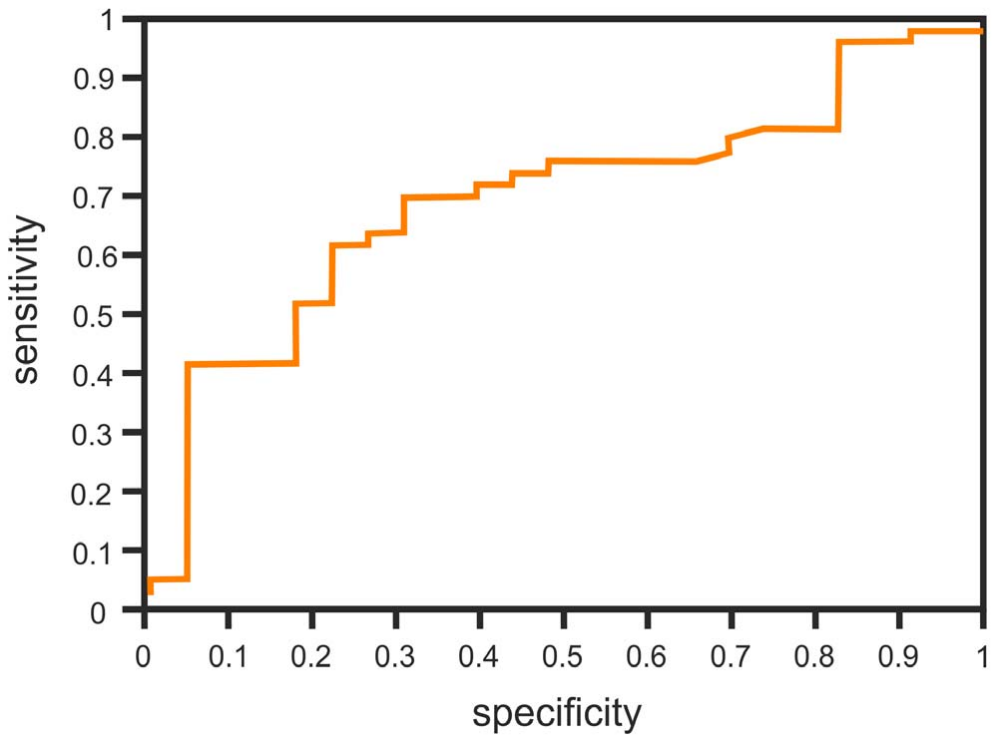
The ROC curve for ΔIL-8.

Logistic regression analysis was performed for the difference of IL-8 concentrations before and after the surgery (ΔIL-8) and for the difference of IL-1β concentrations (ΔIL-1β) in order to determine the independent predictors of PPS. There was a trend for ΔIL-8 concentrations (p = 0.078), leading to a slight increase in the chance of PPS when the value of ΔIL-8 is exceeds 1 pg/ml (hazard ratio 1.009, 95% confidential interval 0.999–1.020). The cut-off point for the best classification variable is 25.2 pg/ml (*Figure 2*). For ΔIL-1β there was no statistical significance (hazard ratio 1.020, 95% confidential interval 0.956–1.088, p = 0.54). Thus, in the logistic regression analysis ΔIL-1β was not an independent predictor of the PPS.

Using the values of the designated cut-off points for IL-8 and ΔIL-8 we calculated sensitivity, specificity and predictive value of both estimated parameters to predict the development of the PPS (*Table 6*).

**Table 6:** The sensitivity, specificity and predictive value determinated by cut-off levels for IL-8 concentration before surgery and the changes of IL-8 concentration (ΔIL-8).

Results	IL-8	ΔIL-8
True negative	18 (24%)	19 (26.8%)
True positive	30 (41%)	31 (44%)
False negative	18 (25%)	17 (24%)
False positive	6 (8%)	4 (5.6%)
All results	73	71
Sensitivity	63%	65%
Specificity	75%	83%
Positive predictive value (PPV)	83%	89%
Negative predictive value (NPV)	50%	53%
Accuracy (Acc)	49%	49%

## Discussion

The cause of PPS is not fully understood. The current hypothesis considers the systemic inflammatory response to be initiated by injury of the pericardial and/or pleural structures, and by the presence of blood within the pericardial and/or pleural space, due to the surgical procedures [9], [21]. It is possible, that the exacerbating factor of the inflammation is the reactivation of a newly infection [9]. The presence of necrotic cells, blood components, and possibly infectious agent stimulates a number of mechanisms for capture and phagocytosis, e.g. neutralization of harmful environmental factors, leading to the regeneration of damaged tissues. The main feature of this inflammation is the presence of the exudate within the serous cavities: pericardial and/or pleural. The inflammatory process involves mainly mesothelial cells and submesothelial connective tissue cells, together forming the lining of the entire surface of the serous cavities.

In the present work, we measured the serum concentrations of IL-1β, IL-6, IL-8, TNF-α as well as IL-10, IL-12p70, before coronary bypass surgery and on the day 5 after the surgery. This particular set of cytokines was chosen based on the following considerations: a) IL-1β, IL-6, IL-8, and TNF-α are the major pro-inflammatory cytokines released from phagocytic cells, b) IL-12 is an important cytokine released from antigen-presenting cells, able to induce of Th1-associated inflammatory responses but also involved in autoimmune tissue destruction. c) IL-10 is important inhibitor of inflammation, derived from activated T and B lymphocytes and monocytes.

The blood samples were collected on the day before surgery and on the day 5 after the surgery, before the PPS developed. The interval was selected to diminish the surgery influence on cytokines elevation. In the literature, early cytokine elevation (IL-1, TNF, IL-6, IL-8) was reported to occur after surgery, as the response to tissues damage, but it normalized on the 3–5 days after surgery [16]–[19].

In order to unify the study group we have studied only patients with a stable coronary artery disease undergoing coronary artery bypass grafting (CABG).

The analysis of the cytokines concentration revealed increase of all studied cytokines in patients who developed the PPS in the follow-up. However, in patients without the PPS, concentration of most cytokines, except for IL-8 and IL-6 concentrations were normalized on the 5th day after CABG. This may indicate an active inflammatory process, leading consequently to chronic serous inflammation and symptoms of the PPS. The comparative analysis of both groups of patients showed significant differences just for IL-8 and IL-1β (p = 0.006 and p = 0.049 respectively). Both of them increased in the PPS patients. Interestingly lower concentration of IL-8 before surgery was an independent risk factor for the PPS development (p = 0.02).

Increased levels of IL-8 in serum has been reported previously in other cardiovascular diseases mainly related to atherogenesis, after cardiac surgery, in patients with severe heart failure, especially in cardiogenic shock [22]. Although, the most abundant source of IL-8 are monocytes and macrophages, it can, in principle, be produced by every nucleated cell type, including mesothelium, the primary target of cardiac surgery-mediated insult [23], [24]. IL-8 is a prototypical member of the chemokine family of small secretory cytokines being the most potent chemoatractants for granulocytes and monocytes [23]. Observation of transgenic mice overexpressing IL-8 showed an increase in circulating neutrophils with reduced expression of L-selectin on their surface, with a tendency to the accumulation of neutrophils in the microcirculation of lungs, liver and spleen [25]. The role of IL-8 in wound healing has been described, involving its chemotactic activity, and stimulation of fibroblasts to produce collagen, fibronectin and tenascin [23]. IL-8 stimulates the endothelial cells division and angiogenesis [23]. Also the involvement of IL-8 released by mesothelial cells and monocytes in the inflammatory processes within serous cavities, particularly in peritoneum, has been reported [26], [27].

**Figure 3:**
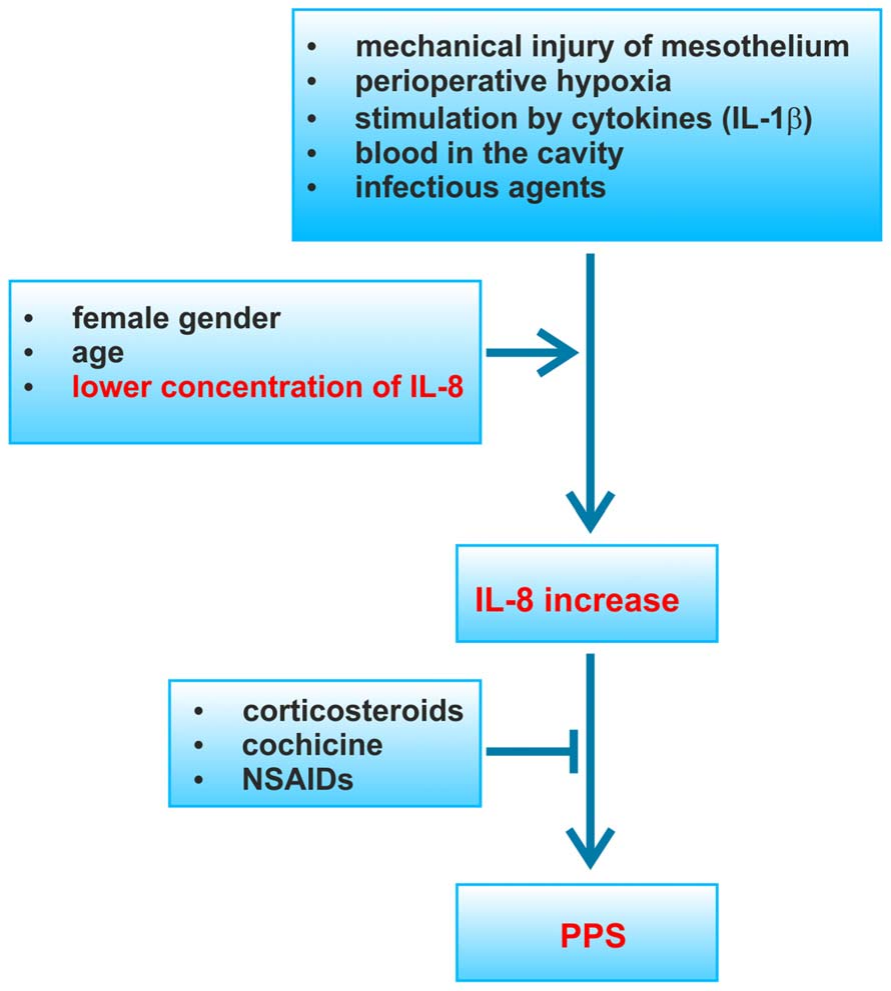
Possible pathogenesis of PPS.

The synthesis of IL-8 is inhibited by glucocorticoids, IL-4, TGF-β inhibitors, 5′lipooxygenases and 1,25 (OH)_2_-vitamin D_3_ [23]. It was noteworthy that aspirin treatment of PPS has an inhibitory effect on the TNF-mediated secretion of IL-8 [22]. Moreover, colchicine, also used in the treatment of the PPS inhibits neutrophil chemotactic response to IL-8 [28]. These data are fully consistent with the observation contained in this report, suggesting an important role of IL-8 in the pathogenesis of the PPS. We postulate that the excessive discharge of IL-8 by mesothelial cells and/or monocytes/macrophages in response to perioperative hypoxia, mechanical injury, stimulation by cytokines (IL-1β), or to the infectious agent(s), induces a change in the phenotype of mesothelial cells to myofibroblasts. Excessive/uncontrolled epithelial-to-mesenchymal transition of mesothelial cells leads to a serosa fibrosis, loss of its physiological role in the regulation of fluid transport and adhesion properties, and to further progression of inflammation and the formation of exudates in serous cavities. At the same time a further stimulation of macrophages and mesothelial cells by the degradation of the glycosaminoglycan layer of apical membrane of mesothelial cells, may stimulate the production of proinflammatory cytokines. Treatment with nonsteroidal anti-inflammatory drugs (NSAIDs), colchicine, or corticosteroids may stop this hypothetical, self-perpetuating mechanism of the PPS pathogenesis (*Figure 3*).

Since none of the several other cytokines showed a similar behavior, we suggested that IL-8 may be specifically involved in the pathogenesis of the PPS, and may have a prognostic value [12]. Unexpectedly, however, the analysis demonstrated that this large increase in IL-8 is not due to higher concentration of the cytokine after the surgery, but because of lower concentration of the cytokine before the surgery, compared to patients without the PPS. Accordingly we found a strongly significant relationship between low IL-8 level before CABG and the risk of developing the PPS. The preoperative levels of IL-8, the value of less than 21.1 pg/ml were significant risk factor for the PPS (hazard ratio 0.977, 95% confidential interval 0.957–0.997). One possible explanation of this finding is that PPS patients are usually younger, less burdened with co-morbidities [29]. Elderly patients or patients with other agents that stimulate chronic inflammation and secretion of cytokines, may actually be adapted to an increased level of the cytokines. The second hypothesis is that high pre-operative concentration of IL-8 might actually be protective against the PPS. This idea is consistent with the results of numerous basic studies showing that IL-8 exerts strongly protective effect on the endothelium as well as on the variety of other epithelial cell types [30]–[33]. Since the mesothelial cells covering the pericardium constitute the primary target of the coronary artery bypass-mediated injury [34], the protective influence of IL-8 on these cells (or other pleural cell populations) could potentially explain the positive association between low IL-8 level before the surgery and the risk of the PPS.

Lower concentration of IL-8 before the operation, as well as a large increase in post-surgery concentration of IL-8 (ΔIL-8), may be measurable risk factors of the PPS in patients after CABG. Selection of patients at risk of developing PPS would allow to include prophylactic anti-inflammatory treatment, and to avoid large effusions in serous cavities, cardiac tamponade and constrictive pericarditis. It would be interesting to add IL-8 assessment in the routine exams before surgery. Patients with low pre-operative IL-8 should be monitored strictly, and otherwise patients with pre-operative high IL-8 would avoid strict monitoring after surgery. Previously conducted studies presented an increase in antiheart antibodies (AHA), accompanying PPS, as a PPS marker [13]. However, this is not a specific marker and it is present approximately 2 weeks from the start of PPS [13], [35]. This feature make the use of AHA impossible as an early diagnostic test. In contrast to AHA, concentrations of cytokines may be an early diagnostic marker of PPS, before the onset of symptoms.

All patients with and without the PPS had significantly higher IL-6 concentrations 5 days after surgery. This finding suggest that the increased levels of IL-6, is directly related to the surgery only and not to the occurrence of the PPS [16].

Another analyzed cytokine: IL-1β showed a similar behavior to IL-8. There was a trend for the PPS development in patients with the lower preoperative concentration of IL-1β compared to patients without the PPS (p = 0.056). Patients who had diagnosed the PPS showed a trend to increase of the cytokine levels in comparison to patients without the PPS, whose had the IL-1β concentration comparable or slightly lower in 5th day after the operation than those obtained prior to surgery. Accordingly, the comparison of the difference in IL-1β in 5 days after surgery, compared to pre-operative levels, showed a significant increase of IL-1β in patients with the PPS group (p = 0.049). However, neither: IL-1β concentration before and 5th day after surgery, nor changes in their concentration were independent risk factor for the PPS. The previously performed studies obtained different results of the detection of IL-1β in response to surgical injury [17]–[19]. The maximum concentration of IL-1β was observed in the first day after surgery [17], [18].

In this study, the PPS was diagnosed in 65% of patients, i.e. more frequently than it is given in the literature (from 10 to 40%). The discrepancy may result from the lower threshold for the diagnosis of pleural effusion. Indeed, in this study, the patients showed more frequent presence of fluid in the pleural cavity (56%) than in the pericardium (31%). The frequency of pleural effusion is higher than the frequency reported in the literature. On the other hand, there is little work on the epidemiological analysis of pleuritis during the course of the PPS, and this may be an underestimated problem.

The major study limitation is related to the definition of the PPS. The diagnosis of PPS is based on clinical evaluation, there are no specific tests to determine the diagnosis. One of the commonly accepted diagnostic criteria for PPS is an inflammation of serous cavities, and fever or biochemical characteristics of inflammation (elevated CRP levels, accelerated ESR, leukocytosis) without the presence of systemic or local infection. The criteria used in the definition are characterized by a low sensitivity and specificity, particularly with regards to patients with early PPS which constitute approximately 80% of patients with the PPS [9]. During wound healing slightly elevated inflammatory parameters can coexist [31] and elevated temperature had been reported in about half of patients with elevated inflammation markers. Elevated body temperature up to 38°C was often episodic and transient. This is due to the widespread use of anti-inflammatory agents in the postoperative period as an analgesic therapy. Further elements of the PPS definition were pleurisy and pericarditis with the presence of fluid in the pleural or in the pericardial. For large exudates the PPS diagnosis is uncertain, even if only slightly elevated inflammatory markers and normal leukocytosis. The presence of small amounts of fluid in serous cavities was in 80% PPS [10], often with a slight increase of inflammatory parameters can be difficult to diagnose with precision.

In the available literature, it is believed that the heart post-traumatic syndrome is more common in younger adults, an average of 47+/−20 years of age [29]. In this study, patients with stable coronary artery disease are homogeneous age group and this may be the reason for the lack of relationship the age and the incidence of PPS. Another described risk factor is female gender [9]. There was a similar trend to a higher incidence of PPS in women (75% vs. 63%), but the difference was not statistically significant (p = 0.16). Other known risk factors for PPS: previously injury of pericardium, treatment with corticosteroids, history of past incidents pericarditis [10] did not occur in the study patients. However, we observed a novel risk factor of PPS to be the low hemoglobin levels before CABG (p = 0.01). Hemoglobin after surgery, transfusion of packed red blood cells in the postoperative period were not associated with an increased risk of PPS. Lower preoperative hemoglobin concentration and its relationship with PPS seems surprising. Perhaps, this means that a person predisposed to the development of PPS is less well adapted to the underlying disease. This would be consistent with the linkage of lower concentrations of cytokines and the risk of PPS. Confirmation of this observation requires further study. Type of the cardiac surgery ECC+ or ECC− did not the risk factor of PPS developing.

## Conclusions

1. Patients with PPS showed the increase of IL-8 and IL-1β concentrations in early postoperative period.
2. IL-8 concentration before the surgery was identified as a risk factor of PPS and could be useful in diagnosis of the disease
3. The lower concentration of IL-8 before surgery and the lower hemoglobin concentration before surgery were the risk factors for the PPS development.
4. Type of cardiac surgery ECC+ or ECC− had no related to the PPS development.
